# Prevalence and novel genetic characteristics of *Cryptosporidium* spp. in wild rodents in the northern foothills of the Dabie Mountains, southeast Henan Province, China

**DOI:** 10.1371/journal.pntd.0013117

**Published:** 2025-05-20

**Authors:** Mengyao Yang, Yin Fu, Pitambar Dhakal, Zi Yan, Jiashu Lang, Chaofeng Ma, Yuhong Jiang, Congzhou Wang, Longxian Zhang

**Affiliations:** 1 College of Veterinary Medicine, Henan Agricultural University, Zhengzhou, Henan, People’s Republic of China; 2 International Joint Research Laboratory for Zoonotic Diseases of Henan, Zhengzhou, People’s Republic of China; 3 Ministry of Agriculture and Rural Affairs, Key Laboratory of Quality and Safety Control of Poultry Products, Zhengzhou, Henan, People’s Republic of China; 4 Technical Service Center for Animal husbandry and Veterinary Medicine of Xinyang, Xinyang, Henan, People’s Republic of China; 5 Technology Exension Center for Animal husbandry and Veterinary Medicine of Shangcheng County, Shangcheng, Henan, People’s Republic of China; 6 Bureau of Agriculture and Rural Affairs of Shangcheng County, Shangcheng, Henan, People’s Republic of China; South China Agricultural University, CHINA

## Abstract

**Background:**

*Cryptosporidium* spp. are prevalent zoonotic pathogens that affect both humans and animals. The pathogens are spread through feces and represent a major cause of diarrhea. As they are both abundant and widely distributed, wild rodents play a significant role in the transmission of *Cryptosporidium* spp. The Dabie Mountains in southeast Henan Province are rich in wildlife resources as well as various species of livestock. However, the epidemiological characteristics of *Cryptosporidium* spp. among local wild rodents remain poorly understood. Therefore, the infection rate and genetic characteristics of *Cryptosporidium* spp. in wild rodents within this region should be determined.

**Methods:**

Between March 2023 and April 2024, a total of 267 wild rodents were captured in the northern foothills of the Dabie Mountains, and fecal samples were collected from their intestines for DNA extraction. Species identification of wild rodents was conducted using PCR amplification of the universal vertebrate cytochrome b (cytb) gene. Nested PCR was subsequently used to amplify the small subunit (SSU) rRNA, actin, heat shock protein 70 (HSP70), and 60 kDa glycoprotein (gp60) genes for the analysis of *Cryptosporidium* species, genotypes, and subtypes in the fecal samples

**Results:**

The infection rate of *Cryptosporidium* spp. in wild rodents from the northern foothills of the Dabie Mountains was 21.3% (57/267). Seven species of wild rodents were identified, and the infection rates for *Cryptosporidium* spp. varied among host species. In particular, the infection rate was 21.4% (25/117) in *Niviventer lotipes*, 22.4% (22/98) in *Apodemus agrarius*, 17.2% (5/29) in *Rattus nitidus*, 22.2% (4/18) in *Apodemus draco*, and 33.3% (1/3) in *Rattus tanezumi*. The identification results indicated the presence of five *Cryptosporidium* species: *Cryptosporidium apodemi* (n = 12), *C. ubiquitum* (n = 11), *C. viatorum* (n = 7), *C. ratti* (n = 2), and *C. occultus* (n = 2). Moreover, two novel genotypes were identified: *Cryptosporidium sp.* rat genotype VI (n = 8) and *Cryptosporidium sp.* rat genotype VII (n = 15). Notably, a new subtype of *C. viatorum* designated as XVgA4 was discovered.

**Conclusions:**

This study revealed the prevalence of *Cryptosporidium* spp. in wild rodents in the northern foothills of the Dabie Mountains and identified two novel *Cryptosporidium* genotypes, along with a new subtype, *C. viatorum*-XVgA4. The findings highlight the genetic diversity of *Cryptosporidium* spp., underscoring the increased risk of *Cryptosporidium* spp. transmission posed by local wild rodents population. It suggests that host-specific factors should be considered in epidemiological surveillance and control strategies of *Cryptosporidium* spp., which is of great significance for the prevention and control of Cryptosporidiosis.

## Author summary

*Cryptosporidium* spp. are zoonotic pathogens that have a significant impact on both human and animal health, commonly causing diarrhea, abdominal pain, and even dehydration leading to death. To investigate the infection rate and genetic characteristics of *Cryptosporidium* spp. in wild rodents of a specific region, we gathered samples from the northern foothills of the Dabie Mountains in southeast Henan Province. A total of 267 rodents were sampled, revealing an infection rate of 21.3%. Notably, we mark the first identification of *Cryptosporidium viatorum* in *Niviventer lotipes* and *Apodemus agrarius*, as well as the detection of *Cryptosporidium occultus* in *Rattus nitidus*. Furthermore, we uncovered two novel *Cryptosporidium* genotypes and introduced a new subtype family within *C. viatorum*. We observed that wild rodents exhibited a higher infection rate of *Cryptosporidium* spp. during autumn and in field habitats, suggesting a close correlation between geographical environment and climate with the transmission dynamics of *Cryptosporidium* spp.

## Introduction

*Cryptosporidium* spp. are important zoonotic pathogens affecting humans as well as domestic and wild animals. The parasites rank as the second leading cause of diarrhea and mortality among children, following rotavirus infection [1,2]. Cryptosporidiosis is primarily transmitted through the fecal–oral route, spreading via contaminated water and food, as well as through person-to-person and human-to-animal contact [3]. As the most abundant and diverse group of mammals, rodents are widely distributed [4] and play an important role in the transmission of various pathogens, including *Cryptosporidium* spp. [5, 6].

The global prevalence of *Cryptosporidium* infection shows considerable variation [7]. At present, the overall global infection rate of *Cryptosporidium* spp. in rodents is approximately 17.0% [8], with prevalence rates ranging from 6.8% to 50.7% [9–16]. To date, at least 31 *Cryptosporidium* species have been identified in rodents [8, 17–19], and 18 *Cryptosporidium* species are known to infect humans. These include *Cryptosporidium hominis*, *C. parvum*, *C. meleagridis*, *C. felis*, *C. canis*, *C. ubiquitum*, *C. viatorum*, *C. mortiferum*, *C. muris*, *C. andersoni*, *C. suis*, *C. scrofarum*, *C. equi*, *C. erinacei*, *C. tyzzeri*, *C. occultus*, *C. ditrichi*, and *C. wrairi* [3]. Notably, *C. viatorum*, *C. muris*, *C. tyzzeri*, *C. ditrichi*, and *C. mortiferum* were initially discovered in rodents [20]. Other common *Cryptosporidium* species identified in rodents include *C. apodemi*, *C. ratti*, and *Cryptosporidium sp.* rat genotypes II-IV [9, 10, 21, 22].

The diverse terrain of the Dabie Mountains in China creates an ideal environment for the survival and reproduction of various flora and fauna, resulting in a region rich in wildlife resources. The Dabie Mountains host a rich diversity of wild rodent species, with variations in species composition across different habitats. In forested areas, the dominant species include *Apodemus draco*, *Apodemus agrarius*, and *Niviventer confucianus*. In agricultural fields and farmlands, the rodent community is primarily composed of species from the genus *Apodemus*, with *A. agrarius* and *A. draco* being the most prevalent. The region also supports a substantial animal husbandry sector.

This study aimed to examine the prevalence of *Cryptosporidium* species among wild rodents in the northern foothills of the Dabie Mountain and to identify the specific species present. Furthermore, we explored the potential zoonotic risks associated with *Cryptosporidium* species carried by wild rodents, thereby providing information for local health authorities for the prevention and control of *Cryptosporidium*. infection in the region.

## Results

### Identification of wild rodent species

Molecular biological identification and analysis identified seven species among the 267 captured wild rodents: *N. lotipes* (n = 117), *A. agrarius* (n = 98), *R. nitidus* (n = 29), *A. draco* (n = 18), *R. tanezumi* (n = 3), *Micromys minutus* (n = 1), and *Arvicola terrestris* (n = 1) (Table 1).

**Table 1:** Infection and distribution of *Cryptosporidium* spp. in wild rodents in the northern foothills of the Dabie Mountains, southeast Henan Province.

Samples	No. positive/no. examined (%)	Species/Genotypes (n)	Subtypes (n)	OR (95% CI)	P
**By rodent species**					0.982
*Niviventer lotipes*	25/117 (21.4)	*Cryptosporidium sp.* rat genotype VII (14), *Cryptosporidium sp.* rat genotype VI (5), *C. viatorum* (5), *C. ubiquitum* (1)	*C. viatorum-*XVgA4 (5)	Reference	
*Apodemus agrarius*	20/98 (20.4)	*C. apodemi* (12), *C. ubiquitum* (7), *Cryptosporidium sp.* rat genotype VI (2), *C. viatorum* (1)	*C. viatorum-*XVgA4 (1)	1.060 (0.547-2.052)	
*Rattus nitidus*	5/29 (17.2)	*C. ratti* (2), *C. occultus* (2), *Cryptosporidium sp.* rat genotype VI (1)	/	1.304 (0.452-3.765)	
*Apodemus draco*	4/18 (22.2)	*C. ubiquitum* (3), *C. viatorum* (1)	*C. viatorum-*XVgA4 (1)	0.951 (0.288-3.145)	
*Rattus tanezumi*	1/3 (33.3)	*Cryptosporidium sp.* rat genotype VII (1)	/	0.543 (0.047-6.240)	
*Micromys minutus*	0/1 (0)	/	/	0.786 (0.715-0.864)	
*Arvicola terrestris*	0/1 (0)	/	/	0.786 (0.715-0.864)	
**Total**	57/267 (21.3)	*Cryptosporidium sp.* rat genotype VII (15), *C. apodemi* (12), *C. ubiquitum* (11), *Cryptosporidium sp.* rat genotype VI (8), *C. viatorum* (7), *C. occultus* (2), *C. ratti* (2)	*C. viatorum-*XVgA4 (7)		

### Identification of *Cryptosporidium* spp.

Among 267 rodent feces samples, 57 positive samples for *Cryptosporidium* spp. were identified using nested PCR targeting the SSU rRNA, actin, and HSP70 genes. This led to an infection rate of 21.3% (57/267) (Table 1). The infection rate of *Cryptosporidium* spp. among wild rodents varied across seasons, reaching a peak in autumn (35.6%, 37/104), followed by summer (14.9%, 15/101). The infection rates were the lowest in winter (8.3%, 1/12) and spring (8.0%, 4/50) (P < 0.01) (Table 2). The wild rodents captured in fields exhibited a higher infection rate of *Cryptosporidium* spp. (26.9%, 39/145) than those captured in forested areas (14.8%, 18/122) (P < 0.05) (Table 2). Among the wild rodent species, *R. tanezumi* had the highest infection rate of *Cryptosporidium* spp., at 33.3% (1/3), followed by *A. chinensis* at 22.2% (4/18), *N. lotipes* at 21.4% (25/117), *A. agrarius* at 20.4% (20/98), and *R. nitidus* at 17.2% (5/29) (P > 0.05) (Table 1).

**Table 2:** Infection and distribution of *Cryptosporidium* spp. in wild rodents in different factors in the northern foothills of the Dabie Mountains, southeast Henan Province.

Samples	No. positive/no. examined (%)	Species/Genotypes (n)	Subtypes (n)	OR (95% CI)	P
**Season**					<0.01
Spring	4/50 (8.0)	*C. viatorum* (2), *Cryptosporidium sp.* rat genotype VI (1), *C. apodemi* (1)	*C. viatorum-*XVgA4 (2)	6.351 (2.119-19.036)	
Summer	15/101 (14.9)	*Cryptosporidium sp.* rat genotype VII (5), *C. apodemi* (5), *Cryptosporidium sp.* rat genotype VI (2), *C. viatorum* (1), *C. occultus* (1), *C. ubiquitum* (1)	*C. viatorum-*XVgA4 (1)	3.166 (1.605-6.247)	
Autumn	37/104 (35.6)	*Cryptosporidium sp.* rat genotype VII (10), *C. ubiquitum* (10), *C. apodemi* (6), *Cryptosporidium sp.* rat genotype VI (4), *C. viatorum* (4), *C. ratti* (2), *C. occultus* (1)	*C. viatorum-*XVgA4 (4)	Reference	
Winter	1/12 (8.3)	*Cryptosporidium sp.* rat genotype VI (1)	/	6.075 (0.754-48.922)	
**Habitat**					0.016
Field	39/145 (26.9)	*Cryptosporidium sp.* rat genotype VI (8)*, C. ubiquitum* (8), *Cryptosporidium sp.* rat genotype VII (7), *C. apodemi* (7), *C. viatorum* (6), *C. ratti* (2), *C. occultus* (1)	*C. viatorum-*XVgA4 (6)	Reference	
Forest	18/122 (14.8)	*Cryptosporidium sp.* rat genotype VII (8), *C. apodemi* (5), *C. ubiquitum* (3), *C. viatorum* (1), *C. occultus* (1)	*C. viatorum-*XVgA4 (1)	2.126 (1.143-3.954)	
**Host gender**					0.837
Female	31/142 (21.8)	*Cryptosporidium sp.* rat genotype VII (10)*, C. viatorum* (5), *Cryptosporidium sp.* rat genotype VI (4), *C. ubiquitum* (4), *C. apodemi* (4), *C. ratti* (2), *C. occultus* (2)	*C. viatorum-*XVgA4 (5)	Reference	
Male	26/125 (20.8)	*C. apodemi* (8), *C. ubiquitum* (7), *Cryptosporidium sp.* rat genotype VII (5), *Cryptosporidium sp.* rat genotype VI (4), *C. viatorum* (2)	*C. viatorum-*XVgA4 (2)	1.063 (0.591-1.913)	

### *Cryptosporidium* species and genotypes

The sequencing and phylogenetic analysis using the SSU rRNA, actin, and HSP70 genes identified five known *Cryptosporidium* species with zoonotic potential, along with two novel genotypes. The study identified *C. apodemi* (n = 12), *C. ubiquitum* (n = 11), *C. viatorum* (n = 7), *C. ratti* (n = 2), and *C. occultus* (n = 2), along with two novel genotypes named as *Cryptosporidium sp.* rat genotype VI (n = 8) and *Cryptosporidium sp.* rat genotype VII (n = 15).

**Fig 1:**
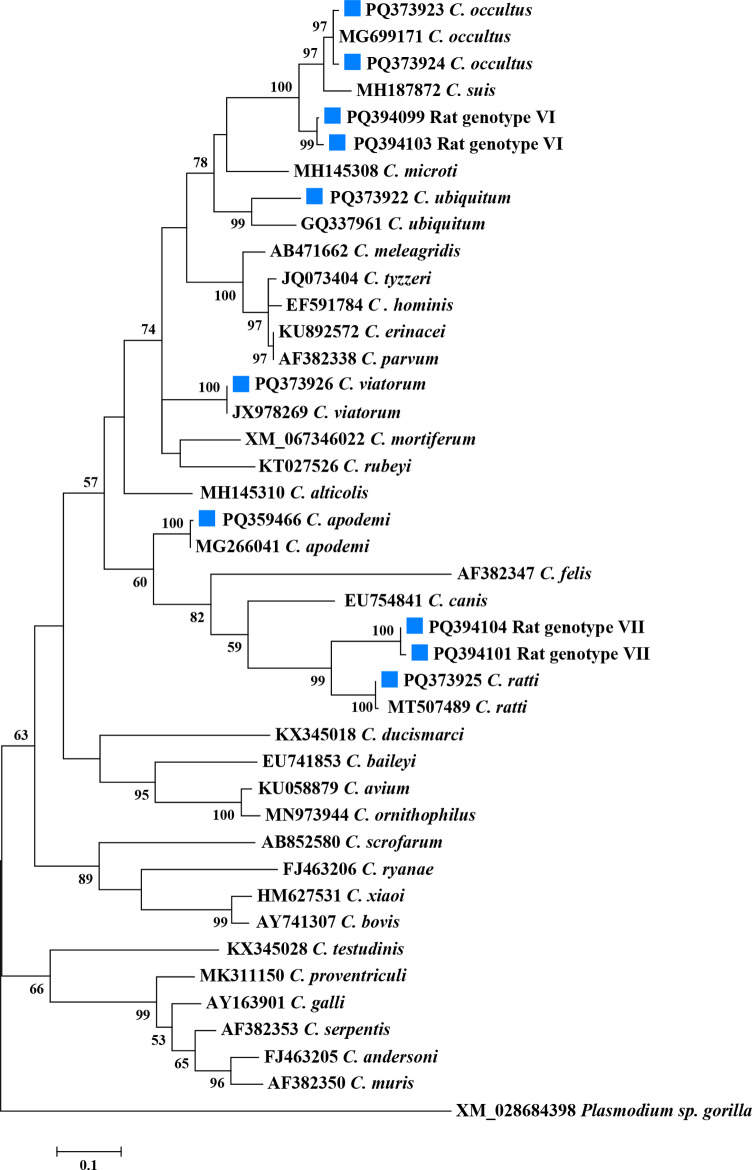
Phylogenetic relationships of *Cryptosporidium* spp. Phylogenetic trees based on the maximum likelihood analyses of the actin gene. Bootstrap values > 50% from 1000 replicates are displayed. The blue squares represent the newly submitted sequences of *Cryptosporidium* species and genotypes identified in this study. The scale bar indicates 0.1 nucleotide substitutions per site.

Among these *Cryptosporidium* species and genotypes, the nucleotide sequences generated from *C. ubiquitum* and *C. apodemi* were identical to the GenBank sequences MT507486 and MG266041, respectively. In addition, the nucleotide sequences from *C. ubiquitum, C. apodemi*, *C. viatorum*, *C. occultus*, *C. occultus*, and *C. ratti* had 1–5 single nucleotide polymorphisms (SNPs) compared to the GenBank reference sequences [MT507486, MG266041, JX978269, MT507491, MT507489]. *Cryptosporidium sp.* rat genotype VI and *Cryptosporidium sp.* rat genotype VII were located on independent branches and were identified as two new genotypes. *Cryptosporidium sp.* rat genotype VI had closer genetic relationships with *C. suis* and *C. occultus*, while *Cryptosporidium sp.* rat genotype VII was closely related to *C. ratti* (Fig 1).

### Subtypes of zoonotic *Cryptosporidium* spp

**Fig 2:**
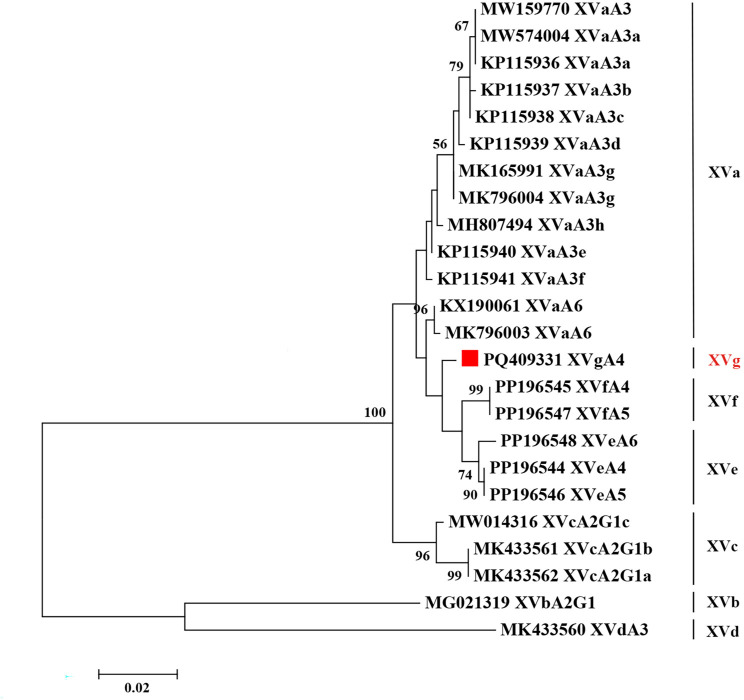
Phylogenetic relationships between various subtypes of *C. viatorum.* Phylogenetic trees based on the gp60 gene sequences of *C. viatorum* were constructed using the maximum likelihood method with 1000 bootstrap replicates. Novel subtypes are indicated by red squares. The scale bar indicates 0.02 nucleotide substitutions per site.

The zoonotic *Cryptosporidium* species *C. viatorum* was further characterized by the gp60 gene. Seven *C. viatorum* samples were successfully amplified. According to the established nomenclature [23], a new subtype family was identified and named XVg. In the phylogenetic analysis, XVg clustered with the known subtype families XVe and XVf and exhibited genetic relatedness to the subtype family XVa (Fig 2). Based on the number of contiguous TCA trinucleotides in the serine repeat region, a new subtype, XVgA4, was identified within the new subtype family.

## Discussion

Wild rodents are widely distributed and have strong reproductive capacity, and they are often in close contact with both humans and domestic animals. The infection rate of *Cryptosporidium* spp. in rodents varies significantly among world regions. In a global meta-analysis, the overall prevalence of *Cryptosporidium* spp. in rodents was 19.8% (4589/23142) [19]. Currently, *Cryptosporidium* infection in rodents have been recorded in 19 countries/regions, with prevalence rates of 0.7%–100%. At 28.0%, Europe has the highest prevalence rate, whereas South American countries exhibit the lowest at 5.0% [8, 19]. Conversely, the infection rate of wild rodents in China varies widely, from 4.0% to 73.9% [24]. The present study is the first to investigate and research the *Cryptosporidium* infection status in wild rodents in the northern foothills of the Dabie Mountains, and the overall infection rate was 21.3% (57/267), which is generally consistent with previous reports.

Several studies have shown that the infection rate of *Cryptosporidium* spp. varies among rodent species. According to previous reports, prevalence rates of *Cryptosporidium* spp. in *Apodemus* spp. range from 13.7% to 31.8% [12, 24], from 21.3% to 22.6% in voles [12, 25], and 14.3% in shrews [12, 22]. In our study, different species of wild rodents exhibited little variation in infection rates. The highest infection rate was observed in *R. tanezumi*(33.3%, 1/3), which was followed by *A. chinensis* (22.2%, 4/18), *N. lotipes* (21.4%, 25/117), *A. agrarius* (20.4%, 20/98), and *R. nitidus* (17.2%, 5/29).

Differences in the distributions of *Cryptosporidium* spp. have been observed among rodent species. *C. parvum*, C. *ubiquitum* and *C. muris* are the predominant species infecting rodents [19]. In the present study, *Cryptosporidium sp.* rat genotype VI, *C. apodemi*, *C. ubiquitum*, and *Cryptosporidium sp.* rat genotype VI showed the highest infection rates. *Cryptosporidium sp.* rat genotype VII was primarily found in rat species such as *N. lotipes* and *R. tanezumi*, while *C. apodemi* exhibited strong host specificity, infecting only *A. agrarius*. *Cryptosporidium ubiquitum* was predominant in *Apodemus* spp., including *A. agrarius* and *A. chinensis*. Furthermore, *Cryptosporidium sp.* rat genotype VI was found in *N. lotipes*, *A. agrarius*, and *R. nitidus*. Nevertheless, we did not find any local wild rodents infected with *C. parvum* and *C. muris*, and the specific reasons warrant further exploration. Notably, this study analyzed the infection rate of *Cryptosporidium* spp. in wild rodents across different seasons. The highest *Cryptosporidium* infection rate was observed in autumn (35.6%, 37/104), followed by summer (14.9%, 15/101). Winter (8.3%, 1/12) and spring (8.0%, 4/50) recorded the lowest infection rate. Previous studies have stated that the infection rates of *Cryptosporidium* spp. in wild rodents generally show little variation between summer and autumn [22, 26, 27]. However, in this study, the infection rate in autumn was significantly higher than in summer. This discrepancy could be due to several factors. The mild temperatures and humid climate in the Dabie Mountains during autumn create favorable conditions for the survival and dispersal of *Cryptosporidium* oocysts. Moreover, wild rodents tend to store large amounts of food and water in autumn to prepare for winter, and this leads to increased activity during this period. These factors may collectively contribute to a significant increase in the *Cryptosporidium* infection rate among wild rodents in autumn.

Furthermore, topography has an impact on the infection rate of *Cryptosporidium* spp. in wild rodents. The sampling sites in this study were primarily divided into field and forest areas. In accordance with previously reported findings, the infection rate of *Cryptosporidium* spp. in wild rodents captured in field was significantly higher at 26.9% (39/145) compared to 14.8% (18/122) in forest areas [22]. The infection rate of *Cryptosporidium* spp. did not differ significantly between male and female rodents.

A total of five *Cryptosporidium* species and two novel genotypes were successfully identified: *C. apodemi*, *C. ubiquitum*, *C. viatorum*, *C. ratti*, *C. occultus*, as well as *Cryptosporidium sp.* rat genotype VI and *Cryptosporidium sp.* rat genotype VII. Among these species, *C. ubiquitum*, *C. viatorum*, and *C. occultus* are known to be zoonotic. *Cryptosporidium ubiquitum*, as an important zoonotic pathogen, can infect a wide range of domestic and wild animals [28]. It has been reported in various wild rodents, such as *A. agrarius*, *A. draco*, *Mus musculus*, and *Rattus norvegicus* [1, 29,30]. This study is the first to report the presence of *C. ubiquitum* in *N. lotipes*, which contributes to a more comprehensive understanding of the ecology and host adaptability of *C. ubiquitum* and provides valuable insights for assessing its zoonotic risks. In China, *Cryptosporidium viatorum* was initially only found in humans and urban wastewater or combined sewage overflow [23, 31–36]. Nevertheless, in 2018, *C. viatorum* was reported in non-human hosts such as *Rattus lutreolus* in Australia, *R. norvegicus*, *Leopoldamys edwardsi*, *Berylmys bowersi*, *Niviventer coninga*, and *A. draco* in China, and *Rattus rattus* in France [1, 37–40]. This study is the first to report that *N. lotipes* and *A. agrarius* can be infected by *C. viatorum*, thereby expanding its host range. This finding also supports the hypothesis that wild rodents may serve as hosts for the zoonotic species *C. viatorum*. Recent studies suggest that the *C. viatorum*-XVa subtype family isolated from wild rodents is genetically similar to subtypes found in humans [40, 41]. The newly discovered XVg subtype family is genetically closely related to the XVa subtype family, although further research is needed. The present study also reports for the first time that *C. occultus* can infect *R. nitidus*. First discovered in rats in 2018, *Cryptosporidium occultus* was later reported in 2020 to infect humans [37, 42]. In addition to the previously mentioned zoonotic *Cryptosporidium* species, *C. apodemi* and *C. ratti* identified in this study have to date only been found in wild rodents, and both of these *Cryptosporidium* species exhibit strong host specificity [21, 24, 43]. The newly discovered genotypes *Cryptosporidium sp.* rat genotype VI and *Cryptosporidium sp.* rat genotype VII were particularly significant in this study, accounting for 23 out of 57 *Cryptosporidium*-positive samples, indicating that these two genotypes may be prevalent among local wild rodents. In the phylogenetic analysis, *Cryptosporidium sp.* rat genotype VI was closely related to *C. suis* and *C. occultus*, while *Cryptosporidium sp.* rat genotype VII was more closely related and belonged to the same clade as *C. ratti*. The Dabie Mountains, which are located in the southeastern region of Henan Province, serve as a natural “gene bank” for species, featuring a diverse array of wild animals and livestock. Cryptosporidiosis has harmed both local public health and animal husbandry. The identification of these two new *Cryptosporidium* genotypes has improved our understanding of the epidemiological characteristics of *Cryptosporidium* spp. in wild rodents in the northern foothills of the Dabie Mountains, providing valuable insights into the development of effective prevention and control strategies in the region.

The infection rate of *Cryptosporidium* spp. in wild rodents is influenced by multiple factors, such as host specificity, geographical distribution, climatic conditions, sampling topography, and host gender [39]. This study revealed that the infection rate of *Cryptosporidium* spp. differed significantly among different seasons and land use forms; however, there were relatively small differences among species of rodents and rodent genders. Therefore, to better understand the epidemiology of *Cryptosporidium* spp. in wild rodents in the northern foothills of the Dabie Mountains, additional sampling of wild rodents within the region is needed.

## Conclusions

This study shows that wild rodents in the northern foothills of the Dabie Mountains, southeast Henan Province, harbor a diverse range of *Cryptosporidium* spp., including five species and two novel genotypes. Notably, this is the first report of *C. ubiquitum* infections *N. lotipes*, *C. viatorum* infections in *N. lotipes* and *A. agrarius*, as well as the identification of *C. occultus* in *R. nitidus*. The discovery of a novel subtype family, XVg, within *C. viatorum* highlights the genetic diversity of these pathogens. These findings emphasize the heightened risk of *Cryptosporidium* spp. transmission posed by wild rodents in the Dabie Mountains. Owing to the large numbers and wide distribution of rodents, the pathogens they carry are a significant source of infection for both humans and domestic animals. Hence, more extensive sampling surveys should be conducted in diverse regions to determine the transmission risk of Cryptosporidiosis from wild rodents.

## Materials and methods

### Ethics statement

All research procedures used in this work were approved by the Institutional Review Board of Henan Agricultural University (approval no. IRB-HENAU-20190820–02).

### Sample collection and processing

**Fig 3:**
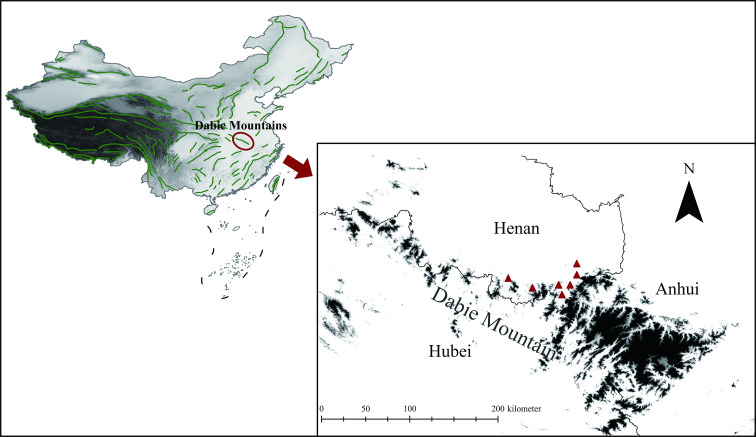
Geographical locations of wild rodents examined in the present study in the northern foothills of the Dabie Mountains, southeast Henan Province, China (red triangle). The map sources are from the National Platform for Common Geospatial Information Services (https://www.tianditu.gov.cn/) and the Geospatial Data Cloud (https://www.gscloud.cn/).

Between March 2023 and March 2024, a total of 267 wild rodents were collected from the northern foothills of the Dabie Mountains in southeastern Henan Province (Latitude range: approximately 31.42°N to 31.90°N. Longitude range: approximately 114.74°E to 115.44°E) (Fig 3 and S1 Table). Featuring a diverse range of landforms, the Dabie Mountains are rich in both wildlife and domestic animal resources. The rodents were captured in rural, field, and forested areas, including farming areas where they frequently interact with domestic animals. After capturing the wild rodents, euthanasia was performed using an overdose of inhaled anesthetic. Subsequently, approximately 500 mg of feces was collected for sampling from the rectal contents of each rodent and stored for subsequent analysis.

### DNA extraction

The whole-genome DNA from wild rodent feces was extracted using a fecal DNA extraction kit (Tiangen Biotech (Beijing) Co., Ltd.) Following the manufacturer’s protocol. DNA was extracted from a total of 267 fecal samples. The extracted DNA was then stored at −20°C for subsequent analyses.

### PCR amplification

Wild rodent species were examined by PCR amplification of the cytochrome b (cytb) gene [44]. *Cryptosporidium* spp. were analyzed through nested PCR amplification of the small subunit (SSU) rRNA, actin, and heat Shock Protein 70 (HSP70) genes [45–47]. *C. viatorum* subtypes were determined using PCR and sequence analysis of the 60 kDa glycoprotein (gp60) gene [23]. Genomic DNA from *Cryptosporidium parvum* (from cattle) and *C. viatorum* (from *Rattus* species) served as positive controls in the PCR analyses.

### Sequencing and phylogenetic analysis

All positive secondary PCR amplicons were sequenced bi-directionally on an ABI 3730 platform by Sangon Biotech (Henan, China). The successfully bidirectionally sequenced DNA fragments were aligned and assembled into complete DNA sequences using Mega 10.0 (http://www.megasoftware.net/). The assembled complete DNA sequences were then subjected to BLAST comparisons, with sequences in the National Center for Biotechnology Information (NCBI) (https://pubmed.ncbi.nlm.nih.gov/) database to identify the species of the parasite. Phylogenetic analysis was conducted using the maximum composite likelihood model, and bootstrap values were calculated by analyzing 1000 replicates. The other parameters were the default values of MEGA 10.0.

### Statistical analysis

Chi-squared tests and 95% confidence intervals (CIs) were calculated using Crosstab in SPSS version 24.0 (SPSS Inc., Chicago, IL, USA). The chi-squared test was used to determine differences in the prevalence of *Cryptosporidium* spp. between rodent species, habitats, seasons, and host genders. The results were considered statistically significant at P < 0.05.

## Supporting information

S1 Table The latitude and longitude information of the wild rodent sampling sites in the northern foothills of the Dabie Mountains, southeast Henan Province. (DOCX)
