# Supplementary material for: Prevalence and novel genetic characteristics of Cryptosporidium spp. in wild rodents in the northern foothills of the Dabie Mountains, southeast Henan Province, China
Source: PLoS Negl Trop Dis. 2025 May 20;19(5):e0013117. doi: 10.1371/journal.pntd.0013117 (PMC12091789; doi:10.1371/journal.pntd.0013117)
Supplement: S1 Table — (DOCX) [file pntd.0013117.s001.docx]

Table S1. The latitude and longitude information of the wild rodent sampling sites in the northern foothills of the Dabie Mountains, southeast Henan Province.

| **Sampling site** | **Site type** | **Latitude and longitude** |
| --- | --- | --- |
| 1 | Field | 31.44°N, 115.27°E |
| 2 | Field | 31.42°N, 115.26°E |
| 3 | Field | 31.51°N, 115.13°E |
| 4 | Field | 31.43°N, 115.12°E |
| 5 | Forest | 31.83°N, 115.25°E |
| 6 | Field | 31.81°N, 115.33°E |
| 7 | Field | 31.82°N, 115.32°E |
| 8 | Field | 31.60°N, 115.34°E |
| 9 | Forest | 31.72°N, 115.40°E |
| 10 | Forest | 31.75°N, 115.46°E |
| 11 | Forest | 31.72°N, 115.41°E |
| 12 | Forest | 31.66°N, 115.36°E |
| 13 | Field | 31.90°N, 115.40°E |
| 14 | Field | 31.81°N, 115.25°E |
| 15 | Field | 31.53°N, 115.30°E |
| 16 | Forest | 31.58°N, 115.34°E |
| 17 | Forest | 31.65°N, 115.36°E |
| 18 | Field | 31.89°N, 115.33°E |
| 19 | Field | 31.89°N, 115.32°E |
| 20 | Field | 31.63°N, 115.36°E |
| 21 | Field | 31.79°N, 115.44°E |
| 22 | Forest | 31.88°N, 115.38°E |
| 23 | Forest | 31.89°N, 115.34°E |
| 24 | Forest | 31.89°N, 115.37°E |
| 25 | Forest | 31.60°N, 114.90°E |
| 26 | Forest | 31.63°N, 114.93°E |
| 27 | Forest | 31.63°N, 114.91°E |
| 28 | Field | 31.60°N, 114.85°E |
| 29 | Field | 31.62°N, 114.74°E |
